# Spontaneous Intracranial Hypotension Presenting as Cryptogenic Coma: A Case Report and Literature Review Emphasizing the Role of Advanced Myelography

**DOI:** 10.7759/cureus.96695

**Published:** 2025-11-12

**Authors:** Jen-Yeu Wang, Tyler E Rice-Canetto, Mohammad Arshad, Louis Reier, Michael Schiraldi

**Affiliations:** 1 Neurosurgery, California University of Science and Medicine, Colton, USA; 2 Neurosurgery, Brooke Army Medical Center, San Antonio, USA; 3 Neurosurgery, Arrowhead Regional Medical Center, Colton, USA; 4 Neurosurgery, Desert Regional Medical Center, Palm springs, USA; 5 Neurosurgery, Riverside University Health System Medical Center, Moreno Valley, USA; 6 Neurosurgery, Redlands Community Hospital, Redlands, USA

**Keywords:** cerebrospinal fluid leak, cerebrospinal fluid venous fistula, craniospinal hypovolemia, meningeal diverticula, spontaneous intracranial hypotension (sih)

## Abstract

Spontaneous intracranial hypotension (SIH) is a difficult-to-diagnose condition that most commonly presents with a postural headache. In this case report we detail an instance of SIH whereby a 57-year-old right-hand-dominant male presented with chief complaint of positional headaches and was found to have a spontaneous right-sided subdural hematoma (SDH). Initially, he was neurologically intact; however, after several days he decompensated and became comatose with anisocoria. A computed tomography scan (CT) of the head showed SDH enlargement and midline shift, prompting emergent craniotomy. He initially improved after surgery, but shortly afterwards developed persistent positional neurological changes; an external ventricular drain revealed low intracranial pressure. Magnetic Resonance Imaging (MRI) revealed diffuse pachymeningeal enhancement, consistent with SIH. CT myelography (CTM) suggested a left Thoracic level 7 CSF leak, while digital subtraction myelography (DSM) identified right Thoracic level 7 and right Thoracic level 10 cerebrospinal venous fistulas (CVFs). Surgical ligation of all three fistulas resolved symptoms initially; however, the patient then experienced rebound intracranial hypertension requiring a ventriculoperitoneal shunt (VPS). At his six-month follow-up appointment, the patient had returned to his neurological baseline, and repeat imaging showed radiographic resolution. This case exemplified several nuances of SIH secondary to CVFs. For one, this is the first case that reports such a severe neurological decline, namely coma. Second, this case adds to the limited existing literature that surgical clip ligation offers definitive treatment. Third, rebound intracranial hypertension can occur following treatment, and patients should be closely monitored for this. Finally, and most importantly, CVFs are a tiny occult pathology that is easily missed and challenging to diagnose but should be considered in cases of non-traumatic SDH. Workup should include both CTM and DSM as these different imaging modalities may reveal different areas of spinal CSF shunting as demonstrated by this case, which we believe is a result of differing flow dynamics.

## Introduction

Spontaneous intracranial hypotension (SIH), also known as craniospinal hypovolemia, was first described by German neurologist Georges Schaltenbrand in 1938 [1]. Diagnosing the condition is challenging and the diagnosis is frequently missed by even the most astute physicians, with an average time of four months from initial presentation to diagnosis [2]. However, with imaging advancements in recent decades, SIH is becoming increasingly recognized. SIH classically presents with postural headache in the setting of low cerebrospinal fluid (CSF) pressure or volume. The annual incidence of SIH is 4-5 per 100,000 population with female predominance, categorizing the condition as uncommon but not rare [3,4].

The pathophysiology of SIH is shunting of spinal CSF secondary to cerebrospinal venous fistula (CVF), spinal meningeal diverticulum, or spinal CSF leak [3,5]. Downward displacement of the brain can result in stretching of the meninges, distortion of cranial nerves, and even brainstem herniation in severe cases. Such displacement can result in nonspecific neurological manifestations such as headache, nausea, cranial nerve deficits, paresthesia’s and even focal weakness [3,6,7]. These changes are reflected in Magnetic Resonance Imaging (MRI) of the brain, with findings summarized by the mnemonic SEEPS: (1) subdural fluid collections, (2) enhancement of the pachymeninges, (3) engorgement of venous structures, (4) pituitary hyperemia, and (5) sagging of the brain [8]. However, given that one-fifth of patients do not have any abnormal findings on brain MRI, SIH is ultimately proven when the CSF shunt is localized on spinal imaging [6,7,9]. Of note, the shunt itself may be as small as the size of a pin and may go undetected on standard Computed Tomography (CT) or Magnetic Resonance (MR) myelography [7,10].

In 2014, Schievink and colleagues published the first radiographic evidence of CVFs on digital subtraction myelography (DSM) [11]. Six years later, they suggested this etiology may account for up to one-fourth of SIH cases [12]. Unique to CVFs, fluid never pools in the epidural space, as it would in the other etiologies. Rather, CSF is shunted directly into the venous system via aberrant communication at a spinal nerve root sleeve, typically into epidural or paraspinal veins. This may be due to underlying connective tissue weaknesses and/or prior CSF hypertension [13]. Up to 20% of patients with spontaneous CSF leaks appear to have skeletal or connective tissue features suggestive of Marfan syndrome or Ehlers-Danlos syndrome, but this association remains to be elucidated specifically in CVFs [14-16]. Treatment for CVFs has traditionally mirrored that of classic spinal CSF leaks: bed rest, epidural blood patch (EBP), fibrin glue, or surgical repair. In 2021, Brinjikji and colleagues reported the first patients treated with transvenous embolization [17]. The endovascular approach, involving catheterization of the implicated paraspinal veins, demonstrated over 90% partial or complete headache response (comparable to surgery) in a recent meta-analysis [18]. However, surgical ligation remains a safe and effective failsafe in refractory cases, as well as an important primary definitive treatment [19,20].

In the current report, we describe the presentation and management of a 57-year-old male patient with three thoracic CVFs, complicated by profound SIH with subdural hematoma (SDH) leading to a drastic neurological decline.

## Case presentation

Case summary

**Figure 1:**
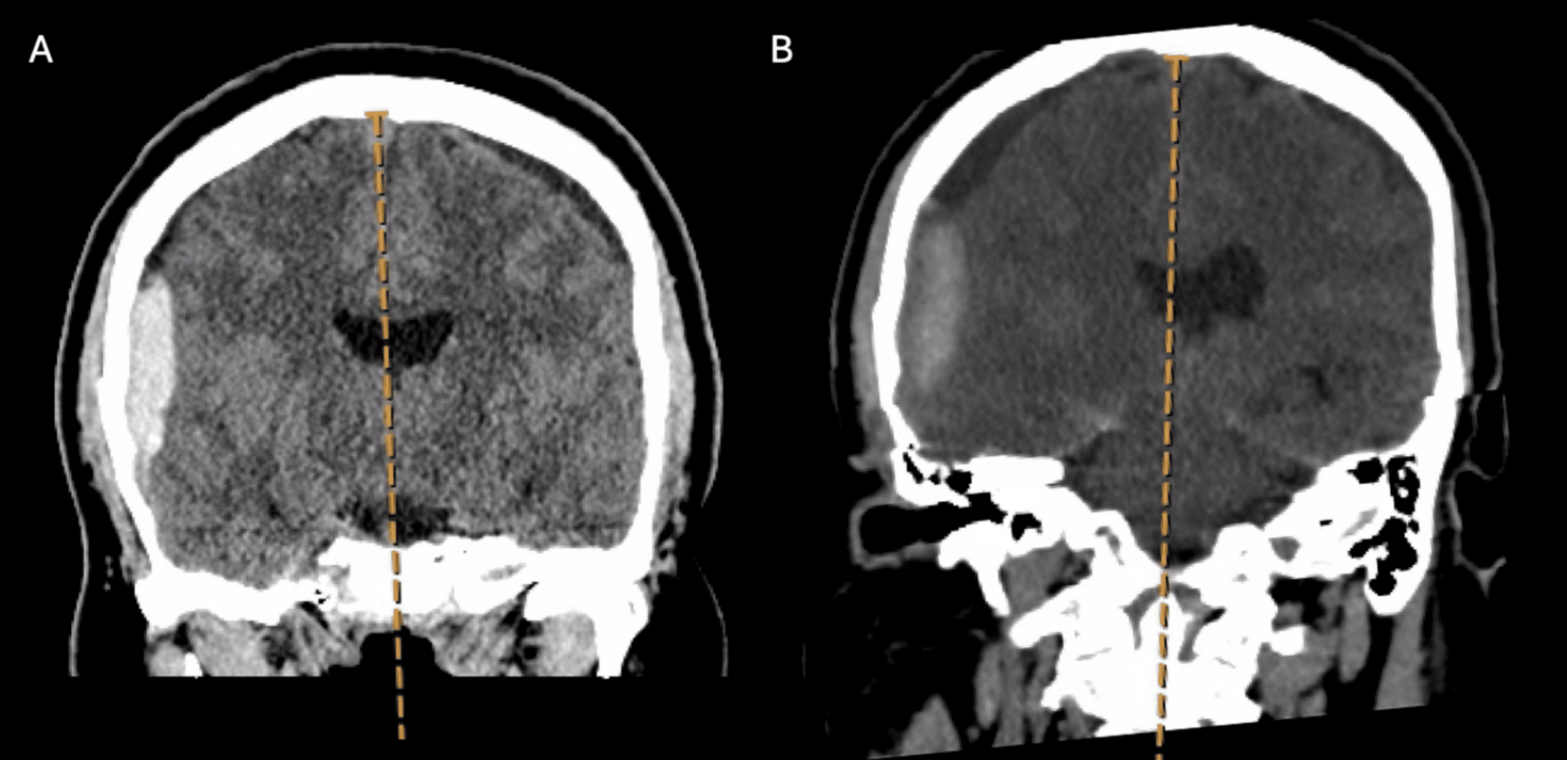
Initial and Early Repeat CT Head A: CT head on initial presentation demonstrating acute right SDH with hygroma present on left side B: Repeat CT head after decline in neurological exam demonstrating increased size of the SDH with mass effect and midline shift CT - Computerized Tomography SDH - Subdural Hemorrhage

A 57-year-old, right-hand-dominant Hispanic male presented to our emergency department with a headache and was found to have a small, right-sided SDH without significant mass effect or midline shift (Figure 1A). The patient denied any recent or remote head trauma. The patient stated that he did have a positional headache that was most severe while in the upright position and improved upon lying down. Several days later patient became somnolent and anisocoric, demonstrating a dilated and sluggishly reactive right pupil. A repeat CT head revealed enlargement of the right-sided SDH and worsening midline shift (MLS) (Figure 1B).

**Figure 2:**
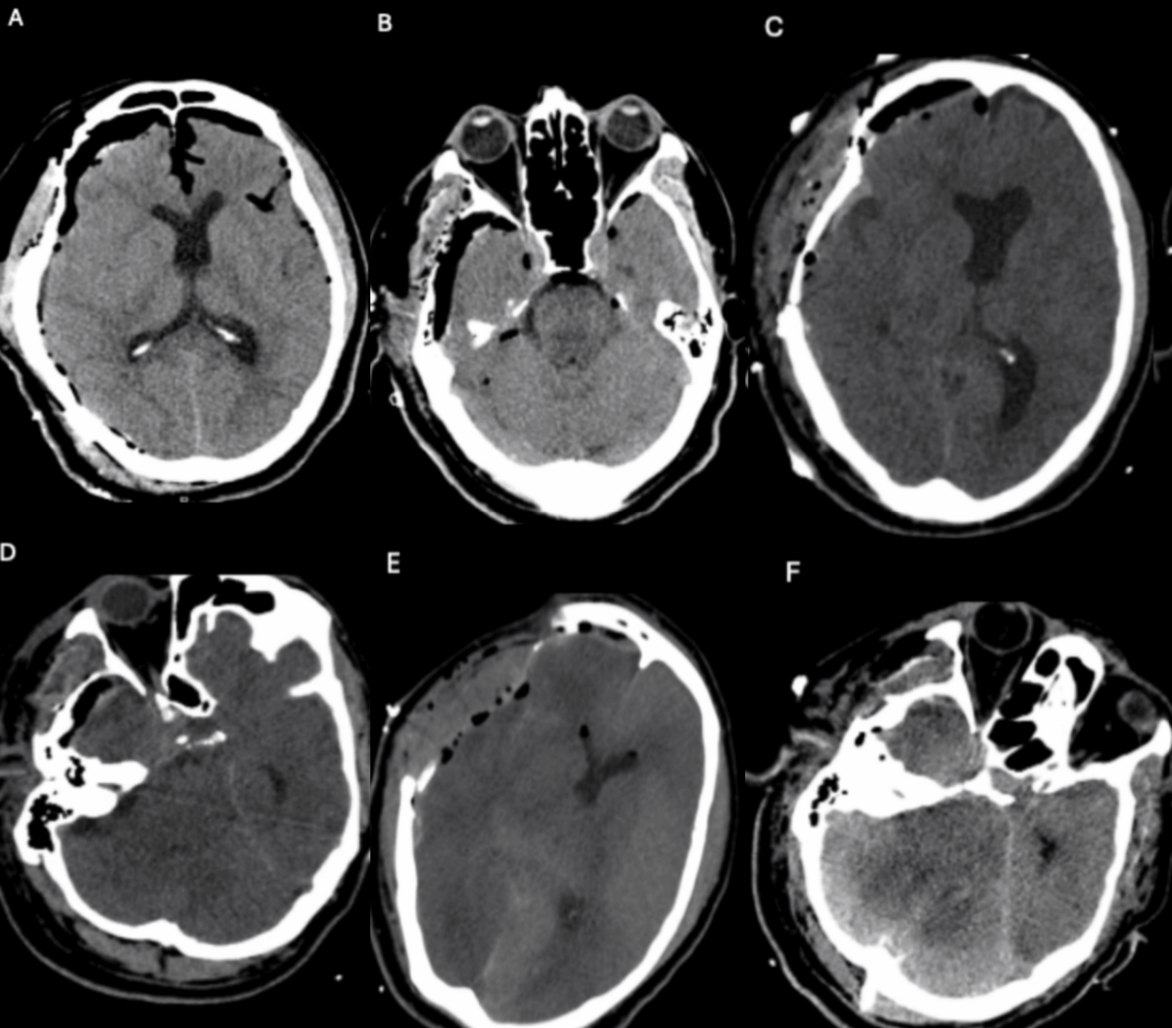
Post-operative and Interval Head CTs A, B: Initial post operative CT head reveals good decompression with no midline shift and pneumocephalus present within the areas of cisternostomy C, D: Interval CT scan the next day at time of patient clinical decline reveals recurring midline shift out of proportion with evidence of brain sag and crowding of basal cisterns E, F: Interval CT head after hemicraniectomy demonstrating improved midline shift, but completely effaced basal cisterns secondary to worsening brain sag CT - Computerized Tomography

The patient subsequently underwent emergent right craniotomy for evacuation of SDH and basal cisternostomy. Immediately after surgery the patient's pupils became equal, but his neurological exam remained otherwise unchanged despite his post-operative CT showing improved MLS (Figure 2A, 2B). The following day, his exam declined, and he again became anisocoric. His right pupil was now fixed and completely non-reactive. Repeat CT head revealed persistent MLS (Figure 2C) and crowding of the basal cisterns (Figure 2D) despite having just undergone SDH evacuation. He was subsequently taken for a right hemicraniectomy but showed no signs of clinical improvement after surgery. CT head post-hemicraniectomy demonstrated improved MLS (Figure 2E) but completely effaced basal cisterns secondary to worsening brain sag (Figure 2F).

**Figure 3:**
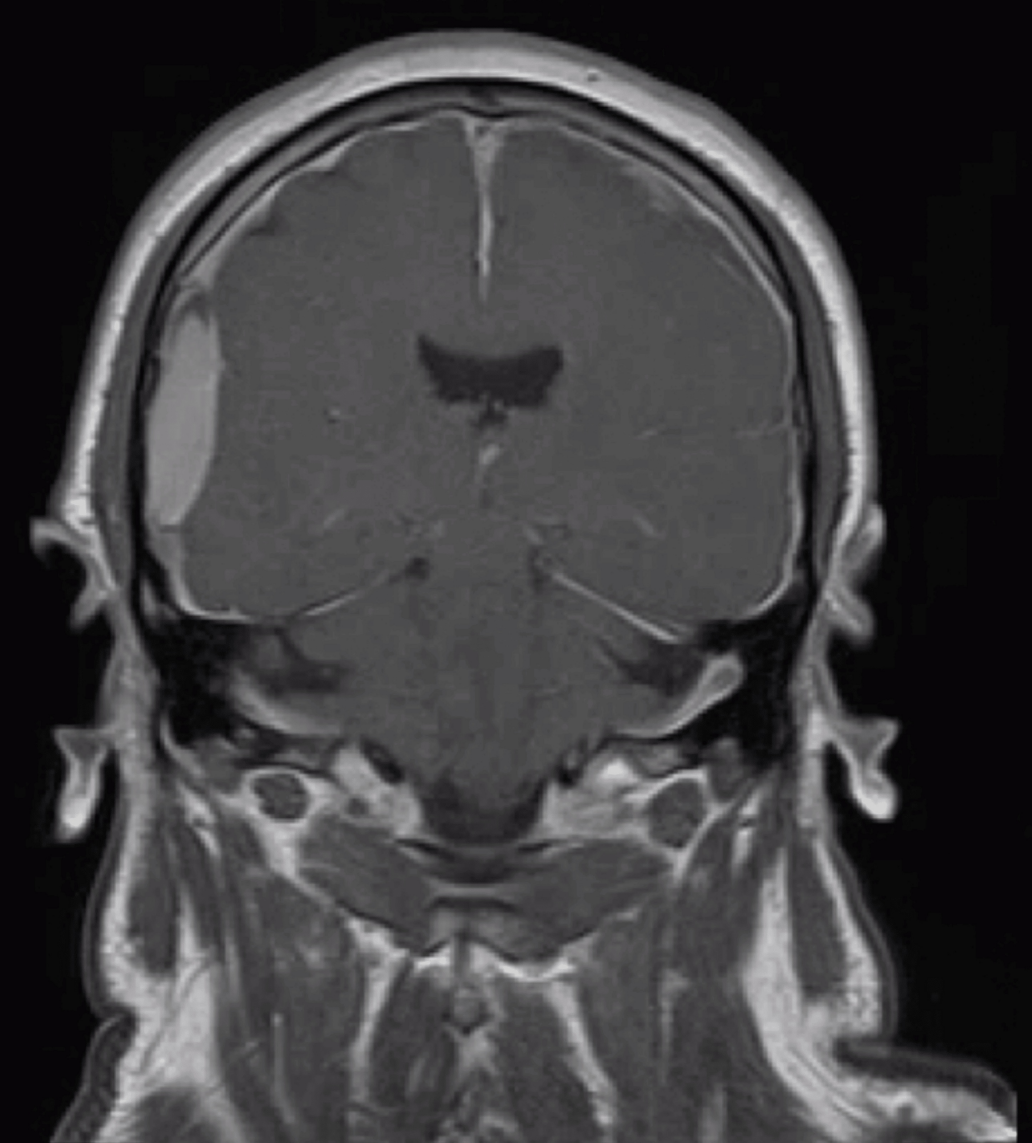
MRI Brain Following Hemicraniectomy MRI brain T1 post gadolinium contrast reveals right SDH with appearance of diffuse leptomeningeal enhancement indicative of spontaneous intracranial hypotension MRI - Magnetic Resonance Imaging SDH - Subdural Hemorrhage

An external ventricular drain (EVD) was subsequently placed, revealing low opening pressure, measuring approximately 1 cm H2O. At this time, it was noted that when the patient was positioned flat, his exam improved, and upon sitting up, his exam declined. A brain MRI with and without contrast showed diffuse pachymeningeal enhancement (Figure 3), and SIH was suspected and worked up further.

**Figure 4:**
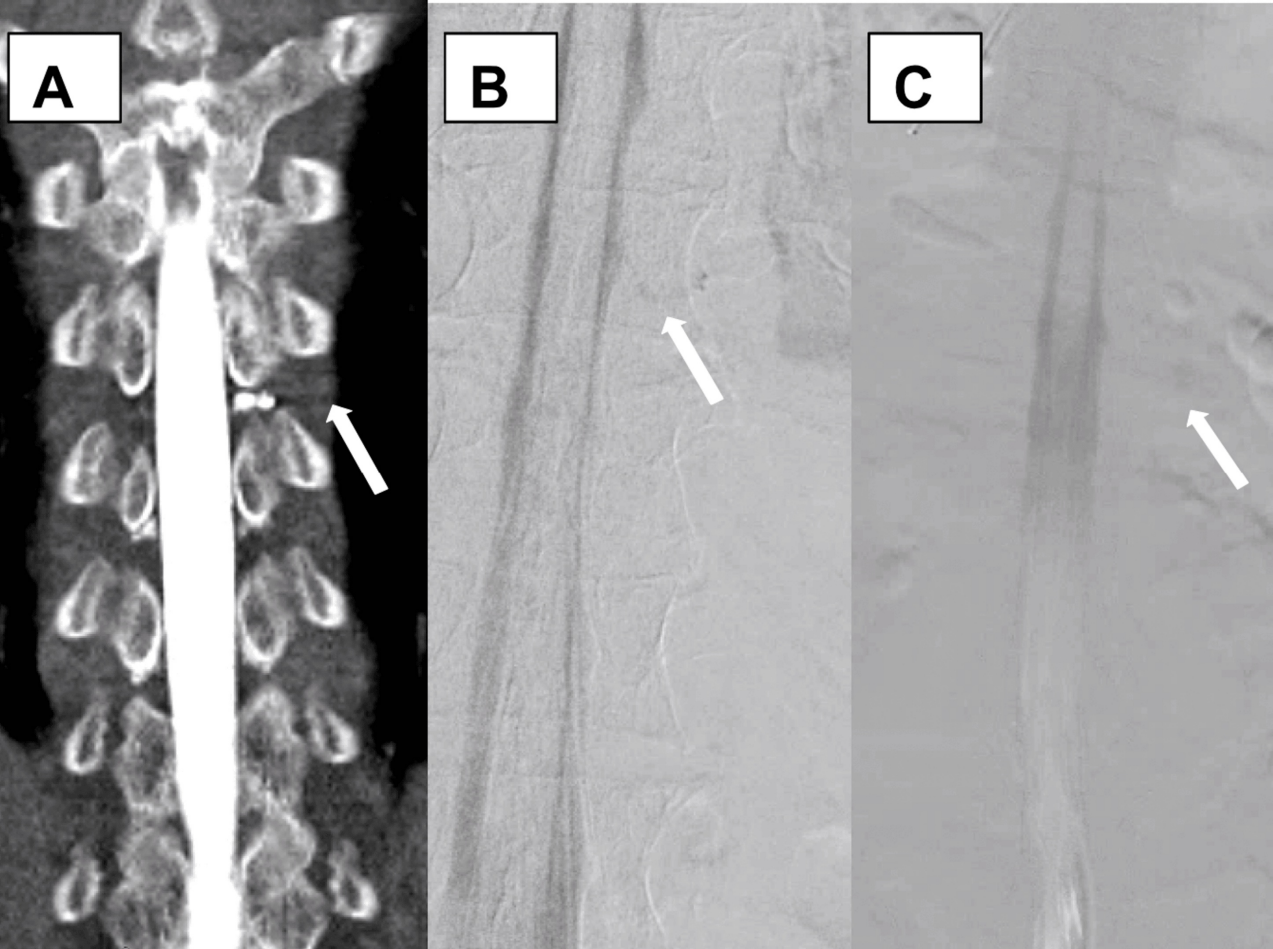
CT Myelography Spine A: Supine CT myelography reveals abnormal signal near left T7 nerve root (arrow) B: Prone digital subtraction myelography reveals CSF-venous fistulas near right T7 nerve root (arrow) C: Prone digital subtraction myelography reveals CSF-venous fistulas near right T10 nerve root (arrow) CT - Computerized Tomography T7 - Thoracic level 7 CSF - Cerebrospinal Fluid T10 - Thoracic level 10

MRI with and without contrast of the entire neuroaxis was unremarkable. CT myelography (CTM) demonstrated an area suspicious for CSF shunting near the left Thoracic level 7 (T7) nerve root (Figure 4A). The patient was treated with epidural blood patch, which did not relieve his positional symptoms. DSM was then completed. Remarkably, DSM did not reveal any abnormality near the left T7 nerve root; however, it did reveal right T7 and right Thoracic level 10 (T10) CVFs (Figure 4B, 4C).

**Figure 5:**
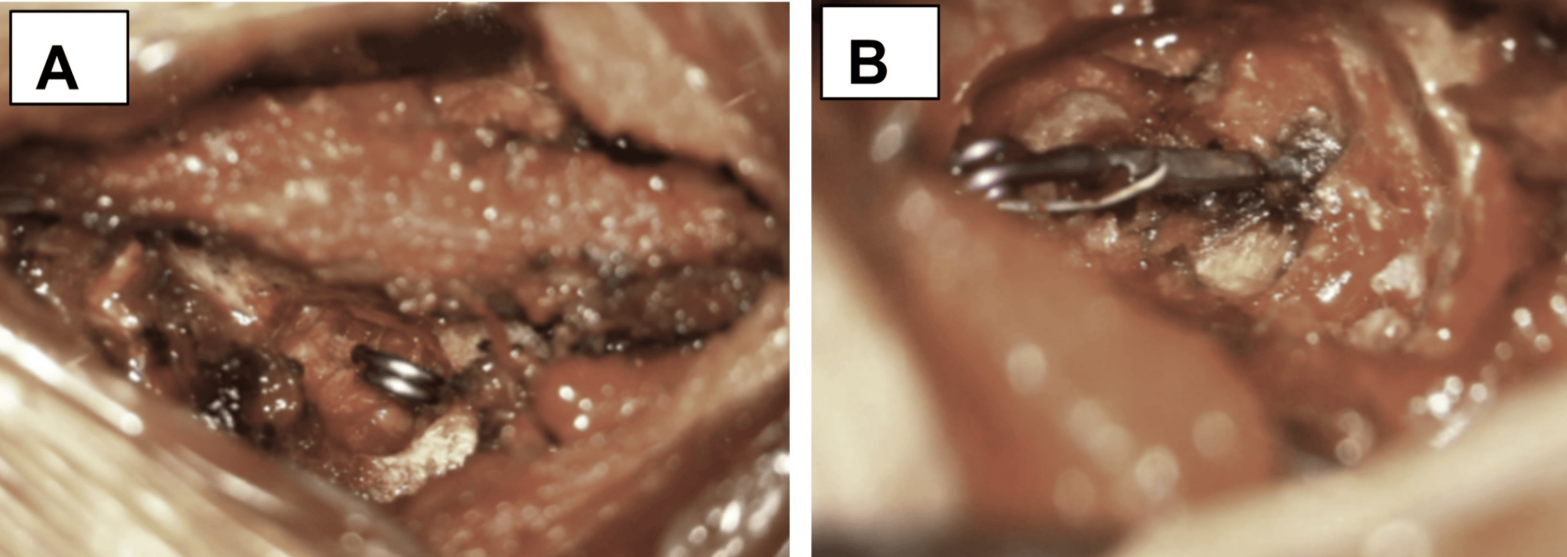
Intraoperative Photos of Extradural Nerve Root Clip Ligation A: Right Thoracic level 7 B: Right Thoracic level 10

The patient then underwent bilateral T7 and right T10 foraminotomy with ligation of all three CVFs (Figure 5).

**Figure 6:**
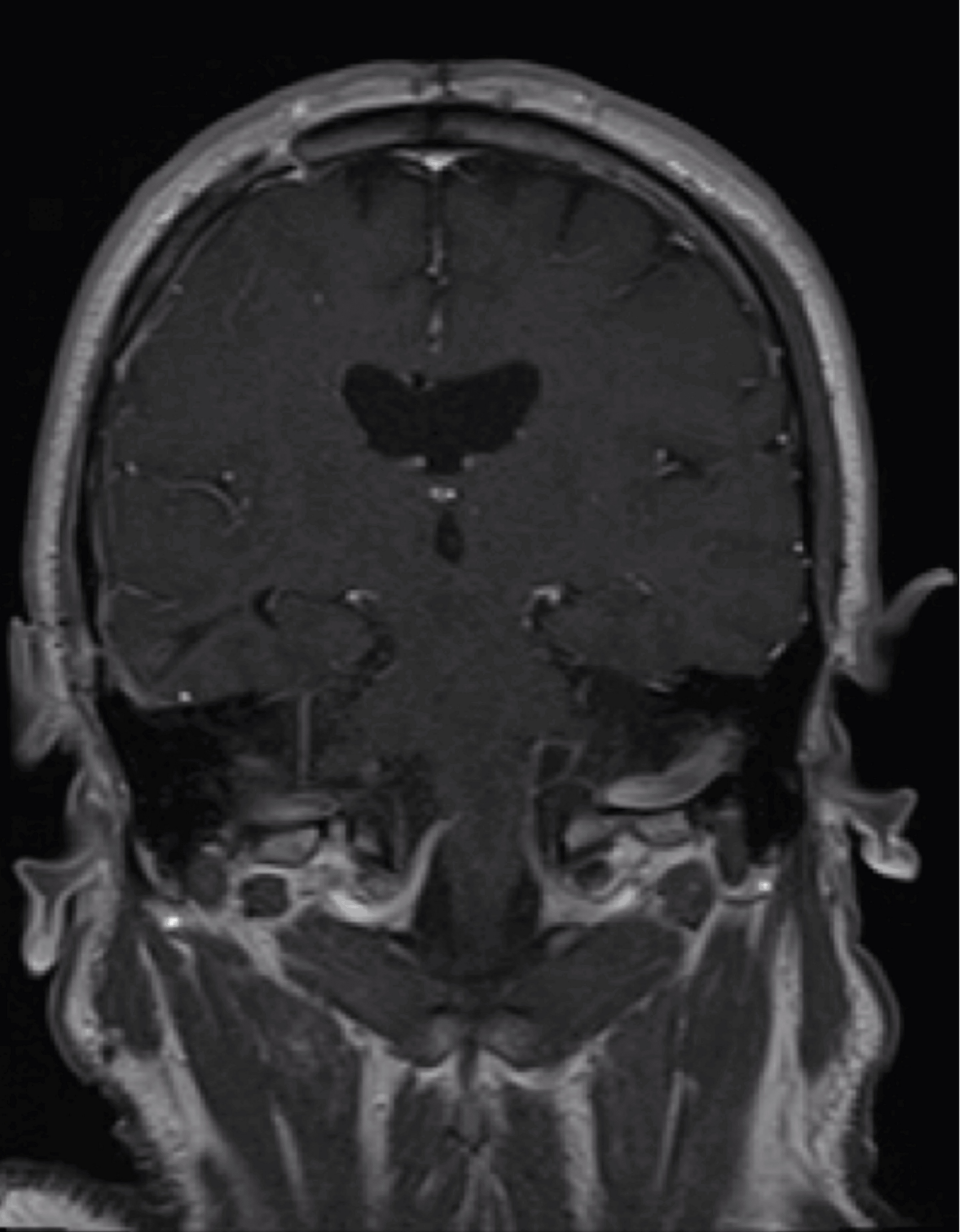
Six-month Interval MRI Brain MRI brain six months after clip ligation of CSF-venous fistulas reveals persistent resolution of subdural hematoma and leptomeningeal enhancement MRI - Magnetic Resonance Imaging CSF - Cerebrospinal Fluid

Following surgical ligation of the CVFs, the patient no longer exhibited positional changes in mentation or anisocoria and was showing signs of rapid clinical improvement. However, his recovery was temporarily impeded by development of rebound intracranial hypertension, requiring placement of a ventriculoperitoneal shunt (VPS). After the VPS was placed, he continued to improve and was discharged shortly thereafter. At his follow-up appointment three months later, the patient was completely neurologically intact, without any residual deficits, positional headaches, or changes in mentation. He was most recently seen at his six-month follow-up appointment and was noted to still be doing well, and at his neurological baseline. He underwent a repeat MRI brain with and without contrast at this time which demonstrated resolution of pachymeningeal enhancement (Figure 6).

Description of subtraction myelography

The patient was positioned prone in standard fashion. The Lumbar level 3-4 interlaminar space was localized under fluoroscopic guidance, and a spinal needle was advanced into the thecal sac, yielding CSF return under low pressure. Approximately 30 mL of contrast was injected gradually over the course of the study. A saline flush was subsequently administered during fluoroscopy to disperse the contrast from the lumbar cistern into the thoracic spine. Digital subtraction imaging demonstrated two areas of abnormal CVFs. 

Description of surgical technique

The patient was positioned prone on a standard open Jackson table. Intraoperative fluoroscopy was used to localize the T7 and T10 vertebral levels. Bilateral T7 and right T10 foraminotomies were performed in standard fashion. At each level, a membranous veil was encountered over abnormally engorged epidural veins and thoracic nerve roots. Without aggressive dissection or stripping of the friable membranes, the thoracic nerve roots were ligated by application of aneurysm clips proximal to the dorsal root ganglion. Patient was informed prior to the procedure of possible intraoperative complications, most notably numbness to the corresponding dermatome or damage to nearby structures including vasculature (artery of Adamkiewicz); the latter of which was mitigated by neuromonitoring. Following clip placement at each level, venous engorgement resolved, consistent with successful occlusion of the CVFs.

## Discussion

SIH is classically associated with orthostatic headaches, cranial nerve deficits, venous sinus thrombosis, and, in some cases, stroke-like symptoms. Our patient presented atypically, with an abrupt neurological decline into a comatose state. Arshad M et al. proposed a grading scale for intracranial hypotension based on presenting symptomatology, scored numerically from 1-3 [21]. Considering this report, we propose a new Grade 4, for patients with SIH who present as comatose (Table 1).

**Table 1:** Updated SIH Grading Scale Proposed updated SIH grading scale based on patient symptomatology, with additional category of Grade 4 SIH - Spontaneous Intracranial Hypotension Permission was obtained from the original publisher to include this modified table in our publication [21]

Grade	Presenting Symptom
1	Positional headache
2	Cranial nerve complaint, spasticity, or hyperreflexia
3	Focal weakness, new-onset seizure, or stroke-like symptoms
4	Comatose state

The literature contains frequent reports of failed treatments and misdiagnoses with regard to SIH [22], which makes sense given that the average time from presentation to diagnosis is four months [2]. To the best of our knowledge, there are no reports of patients progressing to such critical condition in as short a time span as our patient did. Therefore, in cases of non-traumatic SDH, SIH secondary to CVF should be considered and worked up appropriately in a non-delayed fashion.

Spontaneous subdural hematoma with intracranial hypotension

Subdural fluid collections complicate approximately 20-50% of SIH cases because venous engorgement and brain sag place mechanical tension on fragile bridging veins, resulting in subtle stretch and tear [23,24]. Early series by Schievink and colleagues described the broad spectrum of these fluid collections, ranging from thin hygromas to large SDHs causing mass effect [23]. It is apparent that many SIH cases go undiagnosed, particularly in cases caused by CVFs. Therefore, it’s possible that a large proportion of spontaneous subdural hematoma cases, particularly those that recur, may actually be a sequela of undiagnosed CVF.

Localization of spinal venous fistula

Dobrocky and colleagues proposed the Bern score from 0 to 9, determining the likelihood of an SIH diagnosis based on brain MRI [25]. Criteria with corresponding point values are shown in Tables 2, 3, broken up by major and minor criteria. After summing the points based on criteria met, the patient is determined to be low risk if the score is 0 to 2, intermediate risk if the score is 3 to 4, and high risk if the score is 5 or greater. In pursuit of the underlying spinal CSF leak, spine MRI may visualize spinal longitudinal epidural/extradural collections (SLEC) due to pooling of fluid in the epidural space. Schievink and colleagues proposed a classification system based on the mechanism of spinal CSF leak: Type 1 corresponds to dural tears, Type 2 corresponds to meningeal diverticula, and Type 3 corresponds to direct CVFs [5]. Farb and colleagues proposed Type 4 CSF leaks to be distal nerve root sleeve leaks, tracking and dissipating into adjacent facial planes [26]. Table 4 highlights SLEC status and amenability to EBP based on the type of CSF leak present.

**Table 2:** Bern Scale Major criteria <= - less than or equal to mm - millimeters [25]

Major Criteria	Type	Contribution
Engorgement of venous sinuses	Qualitative	2
Pachymeningeal enhancement	Qualitative	2
Supracellar cistern <= 4 mm	Quantitative	2

**Table 3:** Bern Scale Minor criteria mm - millimeters <= - less than or equal to [25]

Minor Criteria	Type	Contribution
Subdural fluid collection	Qualitative	1
Prepontine cistern <= 5 mm	Quantitative	1
Mamillopontine distance <= 6.5 mm	Quantitative	1

**Table 4:** SLEC status and Amenability to EBP based Type of CSF leak Leak SLEC - Spinal Longitudinal Epidural/Extradural Collections EBP - Epidural Blood Patch CSF - Cerebrospinal Fluid [26]

Type	Mechanism	SLEC Status	EBP Treatable
Type 1	Dural tear	Usually positive	Mixed
Type 1a	Ventral	Usually positive	Mixed
Type 1b	Dosrsolateral	Usually positive	Mixed
Type 2	Meningeal diverticulum	Mixed	Likely
Type 2a	Simple	Mixed	Likely
Type 2b	Complex	Mixed	Likely
Type 3	Direct CSF-venous fistula	Negative	Unlikely
Type 4	Direct nerve root sleeve leak	Negative	Unlikely

Conventional spine MRI lacks the resolution required to precisely localize the small CSF shunts usually implicated in SIH. CT or MR myelography, on the other hand, involves the injection of contrast into the thecal sac and is therefore better at identifying small leaks [27]. That being said, subtle leaks still remain challenging and may require DSM for localization. In DSM, real-time tracing of contrast confers the advantage of millisecond temporal resolution, providing the requisite dynamic detail to detect CT or MR myelogram occult leaks.

Our report demonstrates that in some cases of severe SIH, there may be multiple locations of CSF shunting. It also illustrates that different modalities may pick up different types of shunting as the left T7 lesion was only identified on CTM while the right T7 and right T10 lesions were only identified on DSM. We hypothesize that the flow dynamics of the CSF shunt may predict which imaging modality it may be visualized on. A low-flow shunt may be visualized on CTM as stagnated contrast may still be present from time of intrathecal injection to completion of the CT scan. On the contrary, we suspect DSM to be more adept at detecting high-flow shunts. Additionally, lateral decubitus subtraction myelography has been shown to have increased sensitivity to subtraction myelography completed in prone position [12]. Therefore, in cases of SIH, screening imaging with CTM or MR myelography should be complemented with the gold standard DSM to account for multiple lesions of different flow rates of spinal CSF shunting that may be occult on one of these imaging modalities but not the other.

Treatment of CSF-venous fistula

In recent years, transvenous embolization has emerged as a minimally invasive and effective alternative for the correction of CVFs. The overall success rate of this intervention ranges from 90-100%, with nearly all patients having improved headache and/or radiologic findings of pachymeningeal enhanacement and brain sag [17,28]. CT-guided fibrin glue injection is an additional minimally invasive treatment option that is currently under investigation. While additional studies are needed, data from Mamlouk et al. revealed 100% improvement on post-treatment MRI, resolution of CSF-venous fistula on post-treatment decubitus CT myelography, and symptom resolution [29]. However, surgical clip ligation of fistula continues to be the definitive treatment. It offers both overall minimal surgical complications and positive patient outcomes with regard to both radiologic and clinical resolution [18,19]. Surgical techniques have described meticulous dissection of nerve root and stripping of the friable membrane that develops around the dural-venous fistula near the nerve root [7]. This can often be a tedious process, which may not be necessary. In our case, we were able to successfully clip all three fistulas and cure the disease without stripping the nerve root off the friable membranes of the fistula based on serial clinical and radiographic follow-up as far as six months from surgery.

Post-treatment course and rebound intracranial hypertension

Rebound intracranial hypertension has been described in up to one-third of patients after treatment of SIH [6]. This was observed in our patient after successful correction of his thoracic CVFs, and the patient ultimately required VPS placement. It is difficult to ascertain if the shunt dependence resulted from the prior placement of an EVD for ICP monitoring or from the correction of the fistulas creating a CSF imbalance that exceeded the brain’s capacity for endogenous CSF reabsorption. Future studies could involve assessment of CSF dynamics to distinguish between either cause in a larger series of SIH patients. There may even be a small subset of patients with latent idiopathic intracranial hypertension, unmasked by the correction of SIH. Nevertheless, CSF divergence is an important consideration for the post-operative course in fistula correction that should be anticipated by the treating clinician.

## Conclusions

CVFs represent a rare but increasingly recognized cause of SIH, often underdiagnosed due to subtle or absent findings on routine imaging. While SIH is classically associated with orthostatic headaches and subdural collections, this case highlights an unusually severe presentation with anisocoria, coma, and rapid neurological decline. CVFs can lead to recurrent or unexplained subdural hematomas, underscoring their clinical importance. Localization is challenging; conventional MRI or CT myelography may miss small or high-flow fistulas, whereas dynamic subtraction myelography (particularly in the lateral decubitus position) provides superior detection. Notably, different flow dynamics may explain why some CVFs are only visible on certain modalities, making complementary imaging essential. Treatment strategies include transvenous embolization and fibrin glue injection as minimally invasive options, with surgical clip ligation as the definitive approach. Clinicians must anticipate rebound intracranial hypertension after fistula closure, which may necessitate CSF diversion.
